# Complete Remission in a Child With Multisystem Inflammatory Syndrome and Stevens-Johnson Syndrome Treated With Infliximab

**DOI:** 10.7759/cureus.37076

**Published:** 2023-04-03

**Authors:** Shamma Lootah, Elaf Alshammari, Jubran Alqanatish

**Affiliations:** 1 Pediatric Rheumatology, King Abdullah Specialist Children Hospital, Ministry of National Guard Health Affairs, Riyadh, SAU; 2 College of Medicine, King Saud bin Abdulaziz University for Health Sciences (KSAU-HS), Riyadh, SAU; 3 Pediatrics, King Abdullah International Medical Research Center (KAIMRC), Riyadh, SAU; 4 Pediatrics, King Abdullah Specialist Children Hospital, Ministry of National Guard Health Affairs, Riyadh, SAU; 5 Pediatric Rheumatology, King Abdullah International Medical Research Center (KAIMRC), Riyadh, SAU

**Keywords:** mucocutaneous, infliximab, stevens-johnson syndrome (sjs), multisystem inflammatory syndrome in children (mis-c), coronavirus disease 2019 (covid-19)

## Abstract

COVID-19, caused by SARS-CoV-2, can present with various dermatological manifestations, including (albeit rarely) severe mucocutaneous manifestations such as Stevens-Johnson syndrome (SJS) and toxic epidermal necrosis. In contrast, multisystem inflammatory syndrome in children (MIS-C) commonly presents with mucocutaneous manifestations. The presentation of SJS in a child with MIS-C deserves increased attention from clinicians because of its potential fatality. Here we describe a 10-year-old boy with a history of exposure to confirmed COVID-19 who presented with fever, bilateral subconjunctival hemorrhage, cracked and red lips, oral ulcers, and generalized hemorrhagic skin lesions with targetoid lesions. Laboratory tests revealed leukocytosis, neutrophilia, lymphopenia, elevated C-reactive protein, sedimentation rate, ferritin, and B-type natriuretic peptide. A skin biopsy revealed patchy vacuolar interface dermatitis with subepidermal edema and superficial and deep perivascular predominantly histiocytic infiltrates with scattered eosinophils, lymphocytes, and neutrophils suggestive of SJS. In addition to supportive treatment, he was treated with IV methylprednisolone, immunoglobulins, and infliximab, after which his symptoms improved and gradually resolved.

## Introduction

Multisystem inflammatory syndrome in children (MIS-C) was first reported in April 2020, a month after the SARS-CoV-2 was deemed a pandemic. MIS-C presents with multiorgan involvement, including fever, conjunctivitis, mucocutaneous findings, cardiac involvement, gastrointestinal involvement, and hypotension, and is linked to SARS-CoV-2 confirmed through polymerase chain reaction (PCR), antigen test, serology test, or exposure to COVID-19 [1]. The cutaneous manifestations of MIS-C are variable, with very few cases describing Stevens-Johnson syndrome (SJS)/toxic epidermal necrolysis (TEN) as a presenting feature. Katlan B et al. and Karimi A et al. described three patients with MIS-C and SJS [2,3]. Despite intensive treatment, the two patients described by Katlan B et al. died [2]. SJS/TEN, a severe and life-threatening mucocutaneous reaction triggered by drugs or infections, manifests as a painful purpuric rash that may blister as the disease progresses and involve the mucous membranes of the eyes, mouth, and genitalia [4]. This case report aims to increase clinicians’ awareness of the potentially lethal combination of these two severe conditions and show favorable outcomes of prompt intervention.

## Case presentation

**Figure 1:**
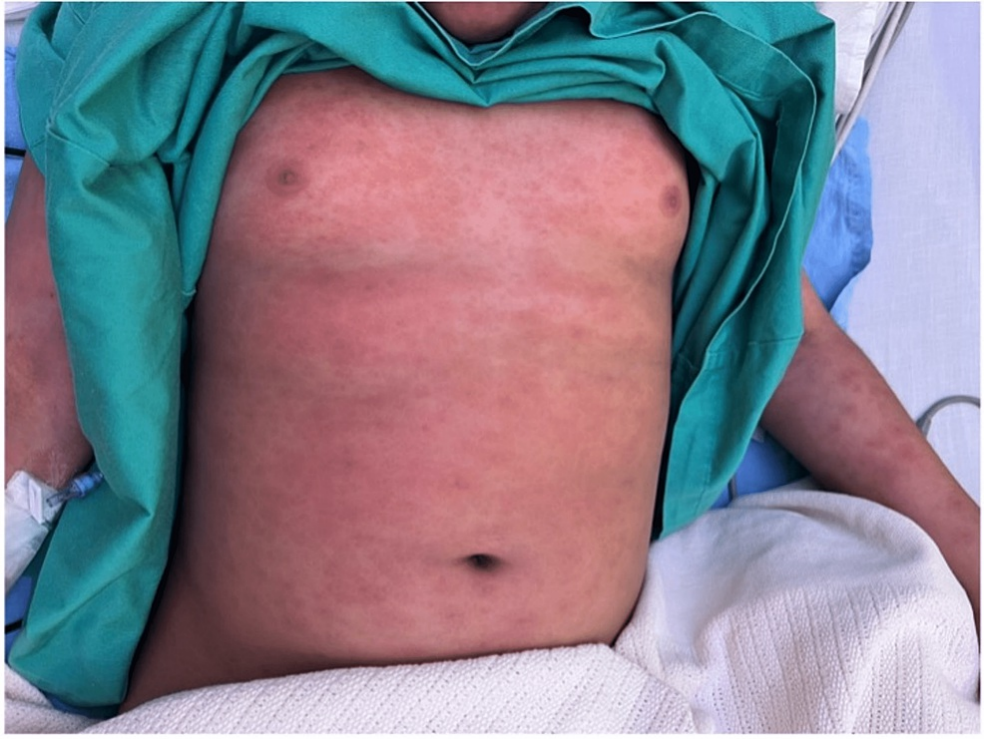
Cutaneous rash.

**Figure 2:**
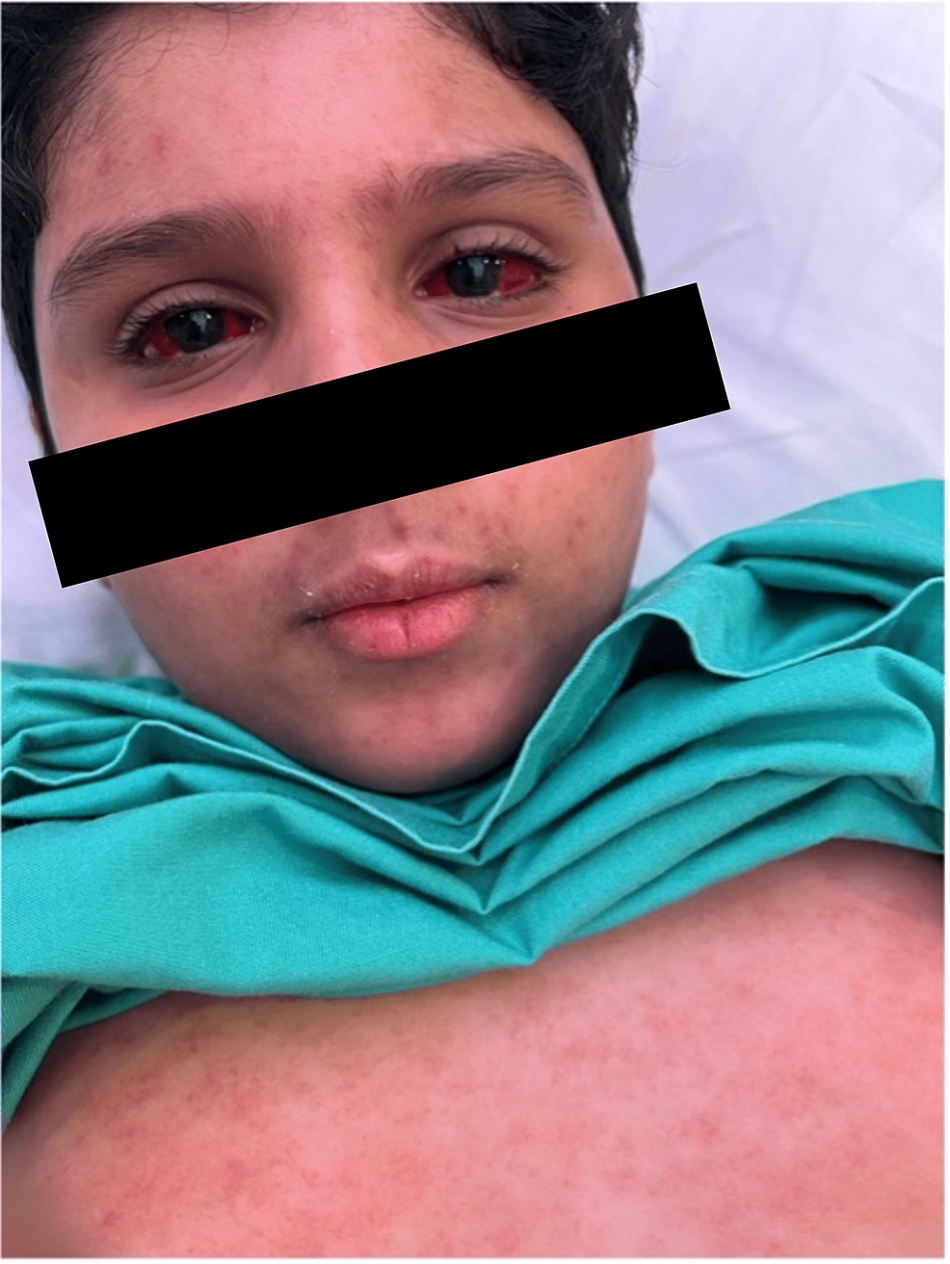
Ocular and oral manifestations.

A previously healthy 10-year-old Saudi boy presented to the ED with a rash that had worsened over the previous five days. He was healthy until he noticed a few small pruritic skin papules over his right distal forearm, hands, soles, cheeks, and mouth. On the third day of the illness, he developed a fever of up to 39°C accompanied by vomiting and general malaise. Other systemic reviews were negative. The patient visited the ED, was prescribed antipyretics (ibuprofen), loratadine, and calamine lotion, and was discharged home with a hand-foot and mouth disease diagnosis. However, on the fourth day of the illness, he returned to the ED with persistent fever, bloodshot eyes, and the rash, having become purplish plaques and more generalized, covering his entire body. He had no history of drug hypersensitivity reactions or a similar rash. He had a history of exposure to COVID-19. He had no history of recent travel or animal contact. His immunizations were up to date, although he had not received the COVID-19 vaccine or the annual influenza vaccine. On examination, he was febrile (temperature 39.1°C), his blood pressure was 95/47 mmHg, his heart rate was 138 beats/min, and his oxygen saturation was 99% on room air. His weight was in the 75th-90th percentile for his age, and his height was in the 10th-25th percentile for his age. He had generalized hemorrhagic skin lesions involving over 90% of the total body surface area, sparing the perineal area. The rash was blanchable and not painful, with occasional target lesions but no bullae or skin peeling (Figure 1). He also had bilateral subconjunctival hemorrhage with normal eye movements and normal cornea and red, cracked lips (Figure 2) with multiple oral ulcers on the tip, lateral sides, and posterior part of the tongue, as well as bilateral palpable submandibular lymph nodes. Chest, heart, and abdominal examinations were normal. 

The patient underwent a set of investigations (Table 1). The complete blood count showed leukocytosis with neutrophilia, lymphopenia, and bandemia. Other labs showed elevated C-reactive protein, erythrocyte sedimentation rate, ferritin, direct hyperbilirubinemia, and coagulopathy. Infections needed to be ruled out with these lab findings and the clinical features. Blood, urine, and throat cultures yielded no growth, and viral workup, including a respiratory viral panel including SARS-CoV-2 and Mycoplasma pneumoniae by PCR, herpes simplex viruses 1 and 2, measles, varicella zoster, and rubella, were negative. With the persistent fever, increasing ferritin, and history of exposure to COVID-19, troponin and brain natriuretic protein were checked, and the latter came back positive. ECG and echocardiogram were also done, and both were normal. Abdominal and renal ultrasounds were done to rule out deep-seated infections, and both were normal. A skin biopsy revealed patchy vacuolar interface dermatitis, scattered dyskeratotic keratinocytes, subepidermal edema, and superficial and deep perivascular predominantly histiocytic infiltrates with scattered eosinophils, lymphocytes, and neutrophils and scattered extravasated RBCs with no evidence of vasculitis.

**Table 1:** Laboratory results during admission and after discharge. BNP: Brain natriuretic peptide; CRP: C-reactive protein; ESR: Erythrocyte sedimentation rate; PTT: Partial thromboplastin time; PT: Prothrombin time; INR: International normalized ratio; AST: Aspartate aminotransferase; ALT: Alanine Transaminase; BUN: Blood urea nitrogen.

	Reference range	Day 0	Day 2	Day 4	Day 10
WBC	4-12 × 10^9^/L	18.80	14.60	15.3	13.2
Neutrophil #	1.1-7.2 ×10^9^/L	13.72	9.93	10.1	4.9
Lymphocyte #	1.3-7.2 ×10^9^/L	0.94	2.34	2.14	6.85
Hgb	113-150 g/L	140	117	127	131
Platelet	150-400 ×10^9^/L	291	175	244	395
Creatinine	27-62 µmol/L	64	49	50	53
BUN	2.5-6 mmol/L	9.0	4.5	4	3.4
Bili D	0-8.6 µmol/L	35.9	8.3	7.3	5.7
Bili T	0-20.5 µmol/L	46.6	13.7	14	15.9
ALT	5-55 U/L	53	78	66	46
AST	5-34 U/L	34	75	50	24
INR	0.8-1.2	1.55	1.2	1.2	1.18
PT	9.38-12.34 s	16.50	13	12	12
PTT	24.84-32.96 s	35.70	33	34	30
ESR	0-15 Mm/h	47	48	30	2
CRP	0-8 mg/L	306	79	31	2
Ferritin	2.1.8-274.6 µg/L	749.2	1125.0	754.2	76.9
Fibrinogen	1.5-4.1 g/L	3.09	2.66	2.45	2.86
D-Dimer	0-0.5 mg/L	8.67	7.41	6.97	0.25
BNP	0-28.9 pmol/L	66.3	68.7	108.2	7.4
Troponin I	0-34.2 pg/mL	9	10	<10	<10

**Figure 3:**
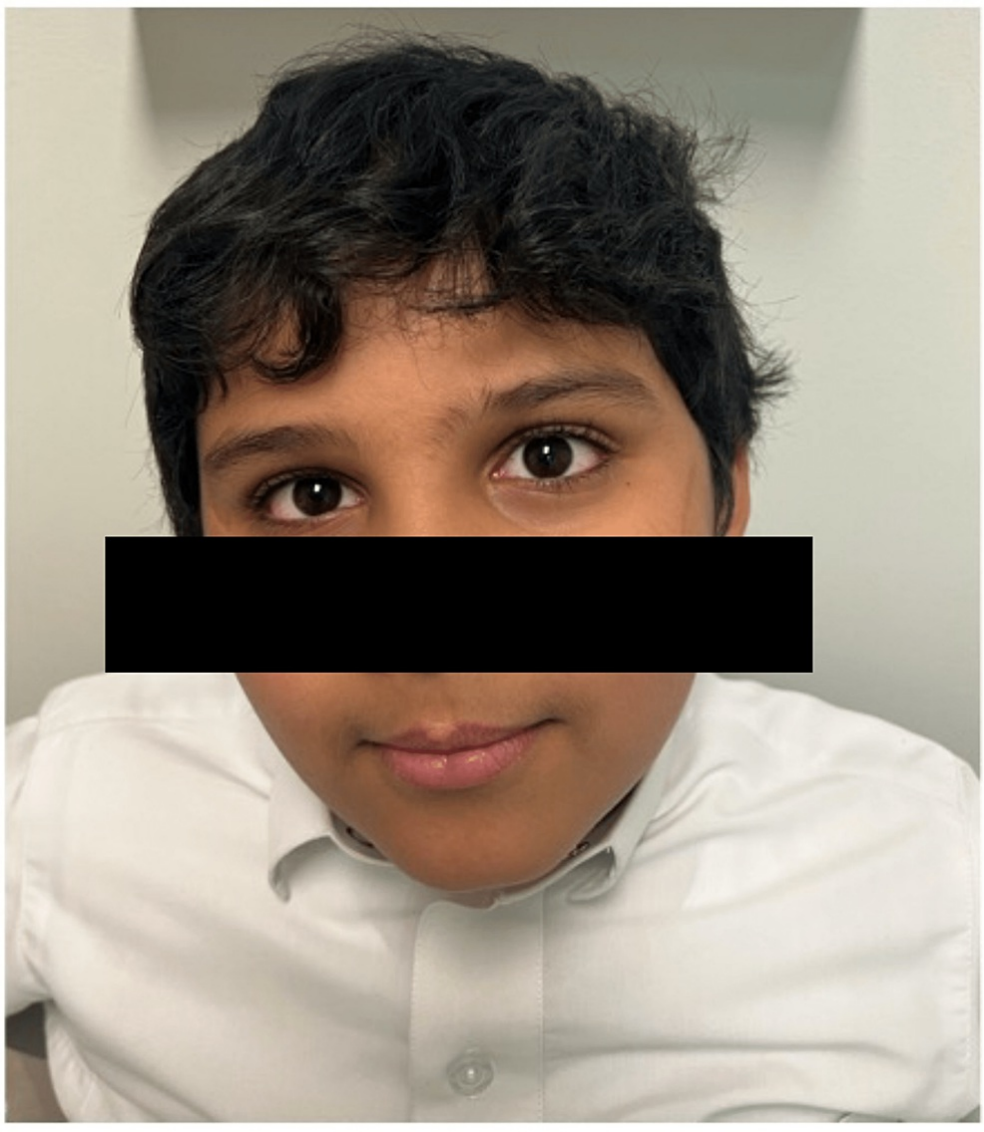
Complete resolution of ocular and mucocutaneous manifestations.

Considering the clinical features of SJS, negative infectious workup, lack of evidence of drugs that might have triggered the rash, and laboratory markers and epidemiological link suggestive of MIS-C, our patient was diagnosed with MIS-C presenting with SJS. The patient was started on intravenous immunoglobulin (IVIG; 1 g/kg) and IV pulse methylprednisolone (30 mg/kg) followed by 1 mg/kg divided over three doses. The patient was covered with ceftriaxone and vancomycin empirically until cultures were negative. For the rash, he was started on topical hydrocortisone 1% and mometasone furoate 0.1% creams; for his eyes, he was prescribed lubricants. His fever subsided, and his eye and mucocutaneous symptoms improved after the IVIG and IV methylprednisolone, but repeated labs revealed increased ferritin levels. Therefore, he received a single dose of IV infliximab (6 mg/kg), after which his symptoms and laboratory markers further improved. He was discharged on the fifth day of the admission on oral prednisone with a tapering schedule, aspirin 81 mg once a day, and topical creams and eye drops. He was seen the week after discharge with much-resolved mucocutaneous symptoms with stable vitals and improved laboratory test findings. He was advised to return for a follow-up in two weeks. At his final visit to the rheumatology clinic, he was doing well clinically, with full resolution of the mucocutaneous manifestations (Figure 3) and normalization of his laboratory parameters (Table 1). Findings of the repeated echocardiogram, which were performed twice after discharge, remained normal; therefore, the aspirin therapy was discontinued.

## Discussion

The cutaneous manifestations of MIS-C have not been well described. Brumfiel CM et al. conducted a retrospective review of 34 case series and reports that identified cutaneous manifestations of MIS-C. Forty-four percent of the analyzed articles used only “rash” to describe the skin lesions, while other articles used more detailed characterizations such as polymorphic, maculopapular, morbilliform, and diffuse erythroderma. Two cases of erythema multiforme and one of leukocytoclastic vasculitis were reported [5]. In a multicenter cohort of children with MIS-C in Saudi Arabia, Al-Harbi S et al. noted rashes in 64.8% of patients, conjunctivitis in 35.2%, and skin peeling in 7.4% [6]. In the pediatric population, MIS-C presenting with SJS has been described in two case reports with different courses and outcomes. Katlan B et al. described two patients who presented with fever and rash confirmed as SJS by a skin punch biopsy and laboratory abnormalities, including leukocytopenia with lymphopenia, elevated acute phase reactants, and impaired liver and kidney function tests. Both patients received broad-spectrum antibiotics, immunomodulators, and plasma exchange. Despite this, they deteriorated, required venovenous extracorporeal exchange, and unfortunately died [2]. Karimi A et al. described a 25-month-old boy with fever and maculopapular rash that started in the feet and then generalized, progressed to bullae, and became ulcerated [3]. He had contact with his mother, who had an upper respiratory tract infection, three weeks prior to becoming symptomatic, and his COVID-19 PCR test result was positive. The patient was treated with steroids, IVIG, and atazanavir. His symptoms improved after these treatments, the follow-up laboratory results were normal, and he was clinically doing well [3]. Similarly, in the current case, the patient received steroids, IVIG, and infliximab, and his condition had dramatically improved on follow-up. 

## Conclusions

Studies of children with MIS-C and SJS are limited. The coincidence of these conditions increases the potential for mortality. Prompt diagnosis and treatment impact outcomes. Here we report the case of an otherwise healthy boy with an unusual presentation of MIS-C with SJS who showed remarkable clinical improvement with a combination of IVIG, steroids, and a single dose of infliximab. By sharing our experience, we hope to enable a higher index of suspicion for the early diagnosis of MIS-C with SJS and change the outcomes of these potentially lethal cases.
